# New methods for estimating the total wing area of birds

**DOI:** 10.1002/ece3.10480

**Published:** 2023-09-02

**Authors:** Hellen (Yi) Fu, Michelle Su, Jonathan J. Chu, Alexandra Margaritescu, Santiago Claramunt

**Affiliations:** ^1^ Department of Ecology and Evolutionary Biology University of Toronto Toronto Ontario Canada; ^2^ Department of Natural History Royal Ontario Museum Toronto Ontario Canada

**Keywords:** dispersal ability, estimation, flight performance, morphometrics, wing area

## Abstract

Dispersal is a fundamental process in evolution and ecology. Due to the predominant role of flight in bird movement, their dispersal capabilities can be estimated from their flight morphology. Most predictors of flight efficiency require an estimate of the total wing area, but the existing methods for estimating wing area are multi‐stepped and prone to compounding error. Here, we validated a new method for estimating the total wing area that requires only the measurement of the wingspan plus two measurements from the folded wings of study skin specimens: wing length and wing width. We demonstrate that the new folded‐wing method estimates total wing area with high precision across a variety of avian groups and wing shapes. In addition, the new method performs as well as the old method when used to estimate natal dispersal distances of North American birds. The folded‐wing method will allow for estimates of the total wing to be readily obtained from thousands of specimens in ornithological collections, thus providing critical information for studies of flight and dispersal in birds.

## Graphical abstract

Estimating flight capabilities from wing shape is a powerful tool for the comparative analysis of dispersal in ecology and evolution. Here, we describe new methods for estimating the total wing area, which will allow for readily estimating wing aspect ratios and wing loadings for thousands of bird species.

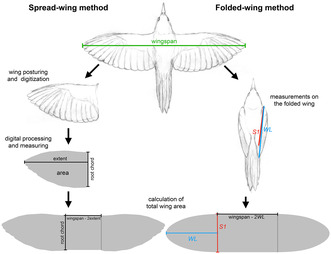

## Resumen

La dispersión es un proceso fundamental en evolución y ecología. Debido al papel predominante del vuelo en el movimiento de las aves, su capacidad de dispersión puede estimarse a partir de su morfología de vuelo. La mayoría de los predictores de la eficiencia de vuelo requieren una estimación del área total del ala, pero los métodos existentes para estimar el área del ala requieren numerosos pasos y son propensos a errores compuestos. En este estudio validamos un nuevo método para estimar el área total del ala que requiere solo la medida de la envergadura y dos medidas de las alas plegadas que pueden tomarse de pieles del estudio: el largo y el ancho del ala. Demostramos que el nuevo método estima el área total del ala con alta precisión en una variedad de grupos de aves y formas de alas. Además, el nuevo método funciona tan bien como el anterior cuando se usa para estimar las distancias de dispersión natal de las aves de América del Norte. El nuevo método permitirá obtener fácilmente estimaciones del área alar total a partir de miles de especímenes en colecciones ornitológicas, beneficiando estudios de vuelo y dispersión en aves.

## 1 INTRODUCTION

As a fundamental process of movement, dispersal can shape the ecology and evolution of birds (Greenwood & Harvey, 1982; Paradis et al., 1998; Winkler et al., 2016). Information about the dispersal capabilities of species can shed light on processes such as population connectivity and demographics, metapopulation dynamics, gene flow, geographic distribution, and speciation (Bohonak, 1999; Claramunt et al., 2012; Gaston, 2003; Martin et al., 2017). Moreover, the capacity to move across heterogenous landscapes may have important implications in conservation by determining the chances of persistence in fragmented habitats and the ability to track optimal environmental conditions that are changing rapidly due to climate change (Lens et al., 2002; MacLean & Beissinger, 2017; Sodhi et al., 2004; Travis et al., 2013). However, very little is known about the dispersal capabilities of most bird species. Banding or tracking devices can be used to study movements but these methods are resource‐intensive and have been used on a very small portion of the global avifauna (Paradis et al., 1998; Weeks et al., 2022).

An alternative approach is to infer dispersal capabilities from the characteristics of the flight apparatus. Since most birds disperse by flying, and flight performance depends on the morphology of the wings, it is possible to quantify the dispersal ability of species from their wing morphology (Claramunt & Wright, 2017; Desrochers, 2010). In particular, there is mounting evidence indicating that birds with more aerodynamically efficient wings (elongated, high aspect ratio) disperse further than those with less efficient wings (Chu & Claramunt, 2023; Claramunt, 2021; Weeks et al., 2022). The morphological characteristic of the wings that affects long‐distance flight efficiency the most is the aspect ratio (Norberg, 1990; Pennycuick, 2008; Taylor & Thomas, 2014). The wing's aspect ratio is calculated with the formula:

(1) $$\text{Aspect ratio}=\frac{{\text{Wingspan}}^{2}}{\text{Total wing area}}$$

in which the wingspan is the maximum extent of both wings measured from wingtip to wingtip on fully extended wings, and the total wing area is the projected surface area of both wings and the intervening body (Pennycuick, 2008). While wingspan measurements have been collected in the past and are reported in research specimen labels, data on total wing area is not available for most species, and preparation of spread‐wing specimens that can be used to estimate wing area has become customary only recently (Claramunt & Wright, 2017; Winker, 2000).

**FIGURE 1:**
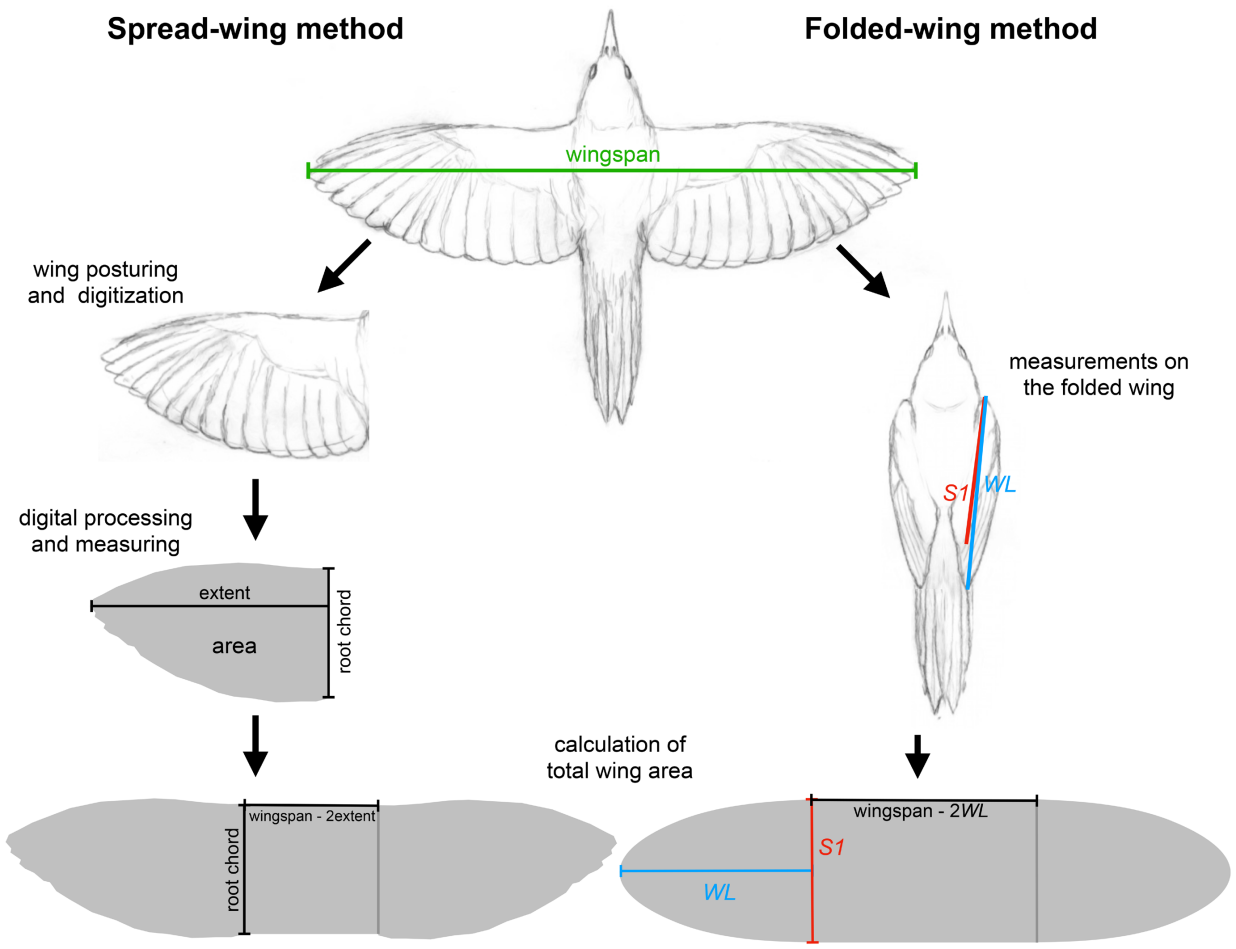
Outlines of the procedures required to estimate the total wing area of birds using the traditional spread‐wing method and the new folded‐wing method. Both methods require the measurement of the wingspan but afterwards, the methods differ. The traditional method requires careful posturing of the wing, digitization using photography, setting the image scale properly, digital processing to make the image binary, single wing area estimation and measuring two additional linear dimensions. The new folded‐wing method only requires two linear measurements taken on the folded wing: the traditional wing length (*WL*) and a similar measurement but to the tip of the first secondary feather (*S1*). See the text and Figure 2 for how these measurements are used to calculate the total wing area.

However, the challenges of estimating total wing area go beyond the limited availability of spread‐wing specimens. Many spread‐wing specimens are not prepared in the standard fully extended position required for evaluating flight performance (Pennycuick, 2008); many are over‐spread or under‐spread. In addition, many spread‐wing specimens do not have an associated wingspan measurement, which is required for the calculations. Finally, the methods commonly used for measuring total wing area require multiple steps during which errors can be introduced (Figure 1). The most traditional method involves tracing the wing outline on grid paper manually and then counting the grid squares and fractions of squares within the outline (Pennycuick, 2008; Stiles & Altshuler, 2004). Alternatively, the wing can be photographed and the area estimated digitally, but distortions can be introduced during photography—mostly due to imprecise alignment between the specimen and the camera, and optical distortions—during image scaling, or when measuring the additional distances required for estimating the total wing area (“extent of wing” and “root chord”, Figure 1), because the exact endpoints of these linear measurements are somewhat arbitrary (Claramunt & Wright, 2017; Pennycuick, 2008). In summary, the current way of measuring the total wing area requires multiple steps and is prone to the accumulation of errors.

Recent alternative methods for calculating wing areas skip the use of extended wings altogether. One method is based on summing the area of individual feathers from feather image databases (Malo & Mata, 2021). Other methods use a few linear measurements from the folded wing to estimate wing area (Figure 1). Wright et al. (2014) described a way of estimating the area of the hand portion of a single wing by assuming that it is shaped as a quarter of an ellipse: the wing length (*WL*, or the distance from the wrist joint to the longest primary feather) would represent the semi‐major axis, and the wing width (*S1*, or the distance from the wrist joint to the tip of the first secondary feather, Eck et al., 2011) would represent the semi‐minor axis, giving rise to the formula *WL*·*S1*·π/4. Claramunt and Wright (2017) found that the formula 3·*WL*·*S1* approximates the total wing area across a large sample of bird groups, but the formula implies that the wingspan is 3·*WL* and the wing is rectangular, two unrealistic assumptions. Finally, Howard et al. (2018) estimated the total wing area by combining wingspan with wing length and wing width in a formula that assumes that the hand‐wing is triangular.

Here, we build on these ideas to develop and validate a new method for estimating the total wing area. The new method only requires data on wingspan, wing length, and wing width, thus not requiring extended wing specimens or images. It combines the use of simple geometric forms for modeling the shape of the hand‐wing (Wright et al., 2014) with the use of the wingspan to estimate the intervening area (Howard et al., 2018). We assessed the precision of the new method relative to the spread‐wing method across a diverse range of species, body sizes, and wing shapes. Finally, to evaluate the efficacy of the new method in studies of dispersal, we used it in descriptors of flight efficiency aimed at predicting natal dispersal distances of North American birds.

## 2 METHODS

### 2.1 Specimens and linear measurements

To compare traditional and new methods on the same individual bird, we used specimens consisting of a study skin with a folded wing, a spread‐wing preparation of the opposite wing, and a measurement of the wingspan (taken prior to the preparation of the study skin). We obtained a total of 112 specimens with these characteristics, 93 from the Royal Ontario Museum (ROM) and 19 from the Louisiana State University Museum of Natural Science (LSUMZ, Appendix 1). Of the former, 88 specimens were newly prepared by the authors using a modification of the standard techniques for preparing study skins (e.g., Blake, 1949; Chapin, 1923) that includes the measurement of the wingspan and the preparation of one of the wings as a separate spread‐wing specimen (Claramunt & Wright, 2017; Winker, 2000). Wingspans were measured by grasping the wings by the metacarpals, stretching them out fully over a ruler, and reading the distance from wingtip to wingtip (the most external primary feather tips along the lateral axis) without flattening the natural curvature of the feathers (Baldwin et al., 1931; Claramunt & Wright, 2017). The spread‐wing preparation followed the standards proposed by Stiles and Altshuler (2004) in which the wing is stretched out and the primaries are spread until the base of the rachis of the outermost primary feather aligns with the leading edge of the proximal wing and becomes perpendicular to anteroposterior axis of the wing (see also Claramunt & Wright, 2017 figure 8.5).

The specimens represented 60 species from 40 families (Appendix 1). The sample included species across the entire range of body sizes for volant birds, from hummingbirds (*Archilochus colubris*, Trochilidae, 3.0 g*)* to one of the largest swans (*Cygnus buccinator*, Anatidae, 13.1 kg), and species spanning the full spectrum of wing morphologies, from an albatross (*Phoebastria nigripes*, Diomedeidae) representing extremely elongated wings (aspect ratio 14.1), a tern (*Sterna hirundo*, Laridae) representing highly pointed wings (aspect ratio 12.1) to the Little Greenbul (*Eurillas virens*, Pycnonotidae) representing short and rounded wings (aspect ratio 4.1). Most species were represented by a single specimen, but we also collected larger samples for three species to analyze how the estimators fare with intraspecific datasets. These three species were selected to represent three different wing shapes: *Cyanocitta cristata* (*n* = 16) representing short, rounded wings, *Columba livia* (*n* = 18) representing intermediate wings, and *Archilochus colubris* (*n* = 17) representing elongated, pointy wings.

We measured wing length (*WL*) and width (*S1*) on the folded wings of standard skin specimens. Measurements were obtained using digital calipers or rulers depending on the size of the specimen. Wingspans were measured by the authors on fresh specimens or obtained from the specimen labels.

### 2.2 Estimation methods

#### 2.2.1 Spread‐wing method

For the traditional spread‐wing method, we used digital images of spread‐wing specimens as follows. Spread wings photographed at the ROM were placed on white paper on a copy stand with a Nikon Z50 mirrorless camera mounted directly above the wing. An L‐shaped ruler was placed next to the wing to provide the scale. Two Godox LEDP260C Bi‐Color LED light panels on tripods were set at 100% brightness and 5000 K color temperature and placed on both sides to minimize shadows. Large wings were photographed in the ROM's photo studio. Wings at LSU were photographed using a hand‐held camera and a digital or manual leveler to ensure the camera was directly above the wing. To reduce measurer error, photographs were taken and digitally measured only by H.F. and M.S.

Wing images were processed and measured digitally using ImageJ v.1.53 (U.S. National Institutes of Health, Bethesda, Maryland). We set the scale using the scale ruler in the image and used the “Threshold” tool to transform the photo into a binary (black and white) image, sometimes followed by the “Fill Holes” option to fill small gaps. Most images were further edited manually using the “Brush” tool to fill small border gaps in broken or disarranged feather vanes, or to fill marginal white spots not distinguished from the background by the thresholding tool. Finally, the “Eraser” tool was used to delete anomalies due to disarranged cover feathers, usually near the shoulder area, and to create a straight proximal border that would represent the root chord (parallel to the axis of the body and perpendicular to the wing leading edge, Figure 1). We then measured the area of the wing using the “Analyze Particles” tool. Finally, using the “Straight Segment” tool, we measured the root chord from the shoulder corner to the trailing edge of the proximal border of the wing, and the extent of the wing from the farthest primary feather tip to the root chord (Figure 1). We then estimated the total wing area using:

(2) $$\text{Total wing area}=2\;\text{Wing}\;\mathrm{area}+\text{Root chord}\;\left(\text{Wingspan}-2\;\mathrm{Wing}\;e\text{xtent}\right)$$

#### 2.2.2 Folded‐wing method

**FIGURE 2:**
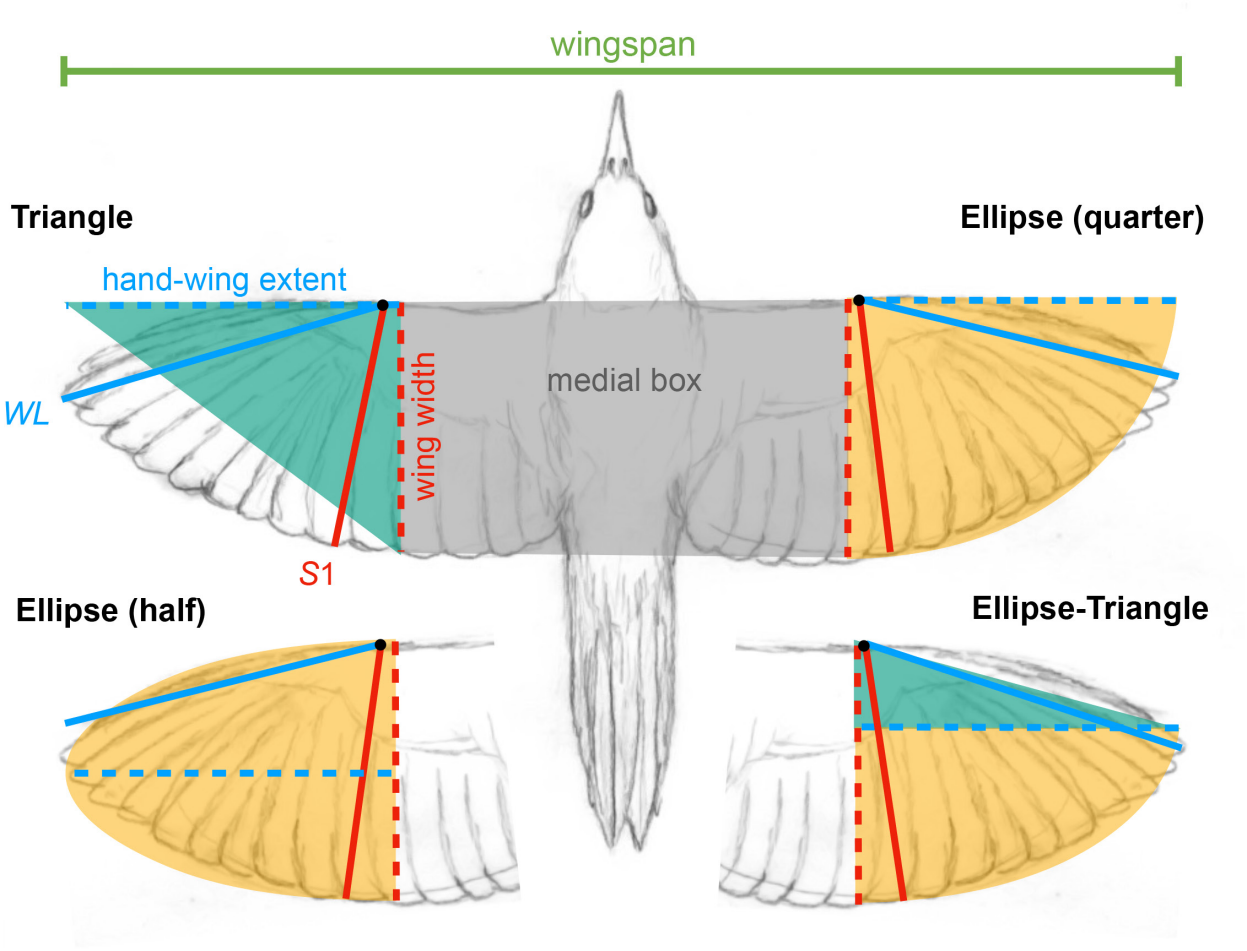
Geometric models used to calculate the total wing area based on wing length (*WL*), wing width (*S1*), and wingspan. The models differ in how the hand‐wing portion of the area is calculated. *WL* is assumed to represent the hand‐wing extent and *S1* the wing width. In the Triangle model, *WL* and *S1* are the legs of a right triangle whose area is calculated as ½*WL*·*S1*. In the half‐ellipse model, *WL* is half the major axis of the ellipse and *S1* is the entire minor axis, whereas in the quarter‐ellipse model *WL* is half the major axis and *S1* is half the minor axis, but both models result in the same formula for the hand‐wing area: ½π·*WL*·*S1* hereafter referred to as the Ellipse model. The Ellipse‐Triangle model combines an ellipse and a triangle in which the ellipse occupies 2/3 of *S1*. See Section 2 for further details.

The method is based on the idea of modeling the shape of the hand portion of the wing as a simple geometric shape whose dimensions and area are determined by *WL* and *S1*, plus an estimation of the area of the rest of the wing and the intervening body (the “medial box”) using the wingspan and *S1* (Figure 2).

The most basic model is the triangle model used by Howard et al. (2018) in which the hand‐wing is assumed to be shaped as a right triangle with *WL* representing the leg corresponding to the hand‐wing extent, and *S1* the leg corresponding to the wing width or chord (Figure 2). The total wing area is then estimated as:

(3) $$\text{Total wing area}\;\left(\text{Triangle}\right)=\frac{\mathrm{WL}\cdot S1}{2}2+\left(WS-2\mathrm{WL}\right)\;S1=\left(\mathrm{WS}-\mathrm{WL}\right)\;S1$$

in which the first summand is the sum of both hand‐wing areas, and the second summand is the area of the medial box, where *S1* is one of the sides of the box and the other is obtained by subtracting two wing lengths to the wingspan (*WS*), analogous to the method used to calculate the root box area in the traditional method (Pennycuick, 2008).

We here propose an Ellipse model. In this model, the hand wing is assumed to have the shape of half of an ellipse (a semi‐ellipse) in which *WL* represents the semi‐major axis (the hand‐wing extent), and *S1* represents the entire minor axis (the wing width, Figure 2, Wright et al., 2014). While this shape would represent wings that are rounded, we also considered assuming the hand‐wing to have the shape of a quarter of an ellipse in which *WL* represents the semi‐major axis as before but *S1* represents the semi‐minor axis (Figure 2). This shape would represent wings that have a straight leading edge and broadly curved trailing edge (or vice versa, Figure 2). However, after some simple algebra, both shapes produce an identical wing area estimator:

(4) $$\text{Total wing area}\;\left(\text{Ellipse}\right)=\frac{1}{2}\pi \cdot \mathrm{WL}\cdot S1+\left(\mathrm{WS}-2\mathrm{WL}\right)\;S1$$

in which the first summand estimates the hand‐wing area, and the second summand estimates the medial box area. Finally, we considered a model that combines a quarter of an ellipse and a triangle, modeling a wing that has an intermediate degree of pointedness between the triangle and the ellipse models (Figure 2). If the two shapes contribute equally, the resultant formula is simply the average of the Triangle and Ellipse models (Equations 3 and 4), but we found that assuming that the elliptical portion is two‐thirds of the shape worked better and resulted in the simple formula:

(5) $$\text{Total wing area}\;\left(\text{Ellipse}-\text{Triangle}\right)=\frac{1}{3}\mathrm{WL}\cdot S1\left(\pi +1\right)+\left(\mathrm{WS}-2\mathrm{WL}\right)\;S1$$

*Aspect ratio*. Using the different estimators of the total wing area, we estimated the wings aspect ratio using Equation (1).

### 2.3 Statistical analyses

For the interspecific analysis, we calculated a measure of discrepancy as the sum of the differences between estimates based on the spread‐wing method and those based on the folded‐wing models (Bland & Altman, 1986); values were log‐transformed (log_10_) to eliminate effects of scaling with body size and the resultant heteroscedasticity. Additionally, we assessed how well the folded‐wing method predicted total wing areas produced by the traditional spread‐wing method using phylogenetic generalized least squares models (PGLS, Freckleton et al., 2002). We used the R package “caper” (v.1.0.1 Orme et al., 2018) in R (v.4.2.0, R Core Team, 2022) to execute PGLS analyses using a lambda correlation structure (Freckleton et al., 2002). The phylogenetic correlation structure was derived from 1000 trees subsetted from a global synthetic tree of all birds (V2.iii, www.birdtree.org, Jetz et al., 2014). The trees were summarized into a Maximum Clade Credibility tree using *TreeAnnotator* (Bouckaert et al., 2014). We fitted regression lines through the origin and interpreted the slope as an indicator of bias (departures from a slope of 1), and the *R* ^2^ as an indicator of relative scatter around the regression lines or precision.

For the intraspecific analyses, discrepancy was assessed by the simple difference between estimates based on the spread‐wing method and those of the folded‐wing models expressed as a percentage (Bland & Altman, 1986). We then assessed precision by calculating coefficients of variation. These coefficients of variation measure overall relative spread, including individual specimen differences and errors introduced in the different procedures used to estimate wing areas. However, because the specimens are the same across methods, any difference in the coefficient of variation would be due to the difference in measuring method.

To evaluate the performance of the new folded‐wing method in a proxy for flight performance, we evaluated the correlation between aspect ratio and natal dispersal distances in North American birds. We used natal dispersal distance estimates from Chu and Claramunt (2023) and added estimates from Martin and Fahrig (2018) for an additional six species to increase the sample of small species: *Cardinalis cardinalis*, *Archilochus colubris*, *Dumetella carolinensis*, *Passerina cyanea*, *Thryothorus ludovicianus*, and *Zenaida asiatica*. We measured *WL* and *S1* in 93 ROM specimens that reported the wingspan in their labels, including several used for the previous analyses, and estimated their total wing areas using the Ellipse model, and the corresponding wing aspect ratio (Appendix 2). We then evaluated the correlation between aspect ratio and natal dispersal distances and compared the results with those obtained by Chu and Claramunt (2023), which used the traditional spread‐wing method. We used PGLS regressions as before and compared models using the Akaike Information Criterion (AIC, Burnham & Anderson, 2002).

## 3 RESULTS

**FIGURE 3:**
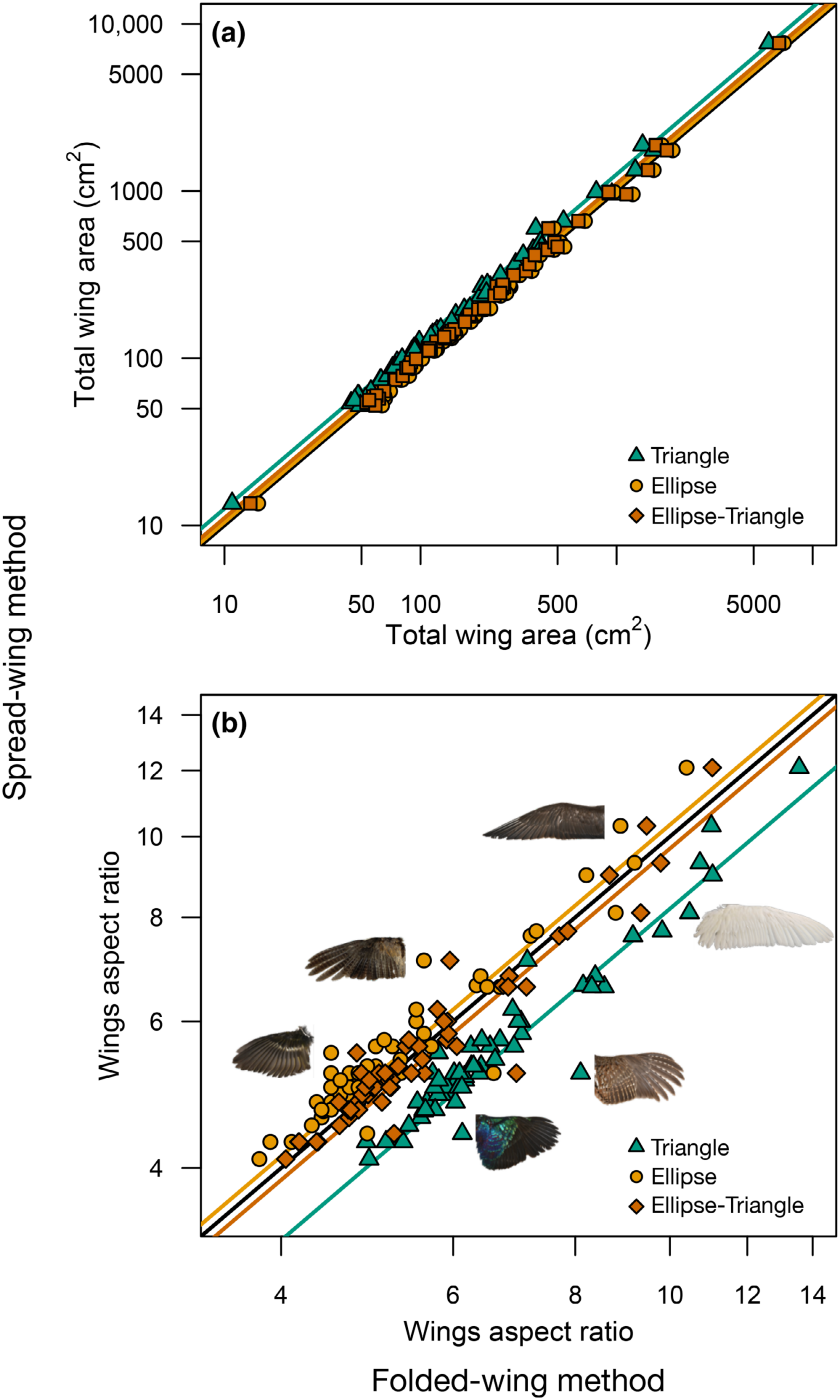
The relationship between estimates of the total wing area (a) and the wings aspect ratio (b) using the traditional spread‐wing method and the new folded‐wing method across 60 bird species. The black line along the diagonal corresponds to the 1:1 equivalence. Colored lines represent phylogenetic regression lines for each geometric model of hand‐wing shape: Triangle, Ellipse, and Ellipse‐Triangle (see Figure 2 and Section 2). Depicted wings in (b) are examples of species that deviate the most from the regression lines (from top to bottom, not at scale): *Stercorarius pomarinus* (ROM 513807), *Cygnus buccinator* (ROM 193625), *Nyctibius griseus* (LSUMZ 192833), *Regulus satrapa* (ROM 513783), *Bonasa umbellus* (ROM 513809), *Lophophorus impejanus* (ROM 513808).

The new folded‐wing method produced estimates of total wing areas that were very close to estimates produced by the traditional spread‐wing method across a wide sample of birds (Table 1, Figure 3a). The Ellipse and the Ellipse‐Triangle models showed more accuracy (low discrepancy, slope closer to 1), while the Triangle model showed slightly higher precision (*r* ^2^ closer to 1, Table 1). When predicting the wings aspect ratio, the Ellipse‐Triangle model showed lower discrepancy, minimal departure from a slope of 1 (as good as the Ellipse model), and nearly maximal levels of precision (barely surpassed by the Triangle model, Table 1, Figure 3b).

**TABLE 1:** Assessment of discrepancy and phylogenetic GLS models of the relationship between the total wing area and the aspect ratio estimated from the folded‐wing method (three models) and the spread‐wing method (the predicted) for 59 species of birds.

Model	Discrepancy	Regression coefficient	Standard error	*r* ^2^	λ
Total wing area					
Triangle	−4.5	1.26	0.011	.995	1
Ellipse	1.5	1.04	0.010	.992	1
Ellipse‐Triangle	−0.4	1.10	0.004	.993	1
Aspect ratio					
Triangle	4.5	0.82	0.021	.920	1
Ellipse	−1.5	1.03	0.029	.909	1
Ellipse‐Triangle	0.4	0.97	0.025	.919	1

*Note*: λ is the lambda parameter of phylogenetic inertia.

**FIGURE 4:**
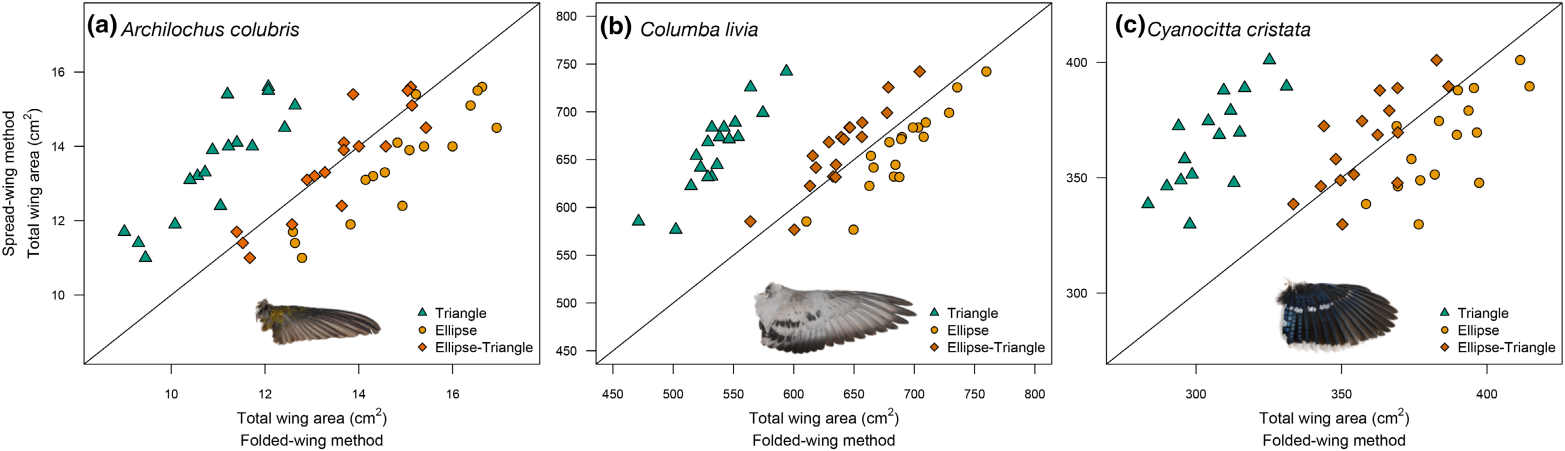
The relationship between total wing areas estimated using the traditional spread‐wing method and the new folded‐wing method in (a) *Archilochus colubris* (Ruby‐throated Hummingbird), (b) *Columba livia* (Rock Dove), and (c) *Cyanocitta cristata* (Blue Jay). The diagonal line corresponds to the equivalence 1:1 line.

In the intraspecific analysis of total wing area, the Ellipse‐Triangle model showed the lowest discrepancy, followed by the simple Ellipse model, across the three species (Table 2, Figure 4). The Triangle model underestimated wing areas by more than 10 percentage points on average. The folded‐wing methods showed lower coefficients of variation than the traditional spread‐wing method for all three species (Table 2).

**TABLE 2:** Statistics of accuracy and precision for estimates of the total wing area of *Archilochus colubris* (*n* = 17), *Columba livia* (*n* = 18), and *Cyanocitta cristata* (*n* = 16) calculated using the spread‐wing method and the new folded‐wing method assuming three different geometries for the shape of the hand‐wing.

Method – Model	Total wing area			
	Mean cm^2^	Difference^a^ (%)	Standard deviation	Coefficient of variation (%)
*Archilochus colubris*				
Spread‐wing	13.5	–	1.47	10.9
Folded‐wing **–** Triangle	11.0	−19.1	1.08	9.8
Folded‐wing **–** Ellipse	14.9	9.8	1.38	9.3
Folded‐wing **–** Ellipse‐Triangle	13.6	0.2	1.28	9.4
*Columba livia*				
Spread‐wing	661.0	–	42.8	6.5
Folded‐wing **–** Triangle	536.3	−18.9	27.3	5.1
Folded‐wing **–** Ellipse	689.4	4.3	33.9	4.9
Folded‐wing **–** Ellipse‐Triangle	638.4	−3.4	31.5	4.9
*Cyanocitta cristata*				
Spread‐wing	365.8	–	20.7	5.7
Folded‐wing **–** Triangle	305.6	−16.5	13.0	4.3
Folded‐wing **–** Ellipse	386.1	5.6	15.4	4.0
Folded‐wing **–** Ellipse‐Triangle	359.3	−1.8	14.6	4.1

^a^ Mean difference between the spread‐wing method and folded‐wing methods as a percentage of the spread‐wing method mean.

**FIGURE 5:**
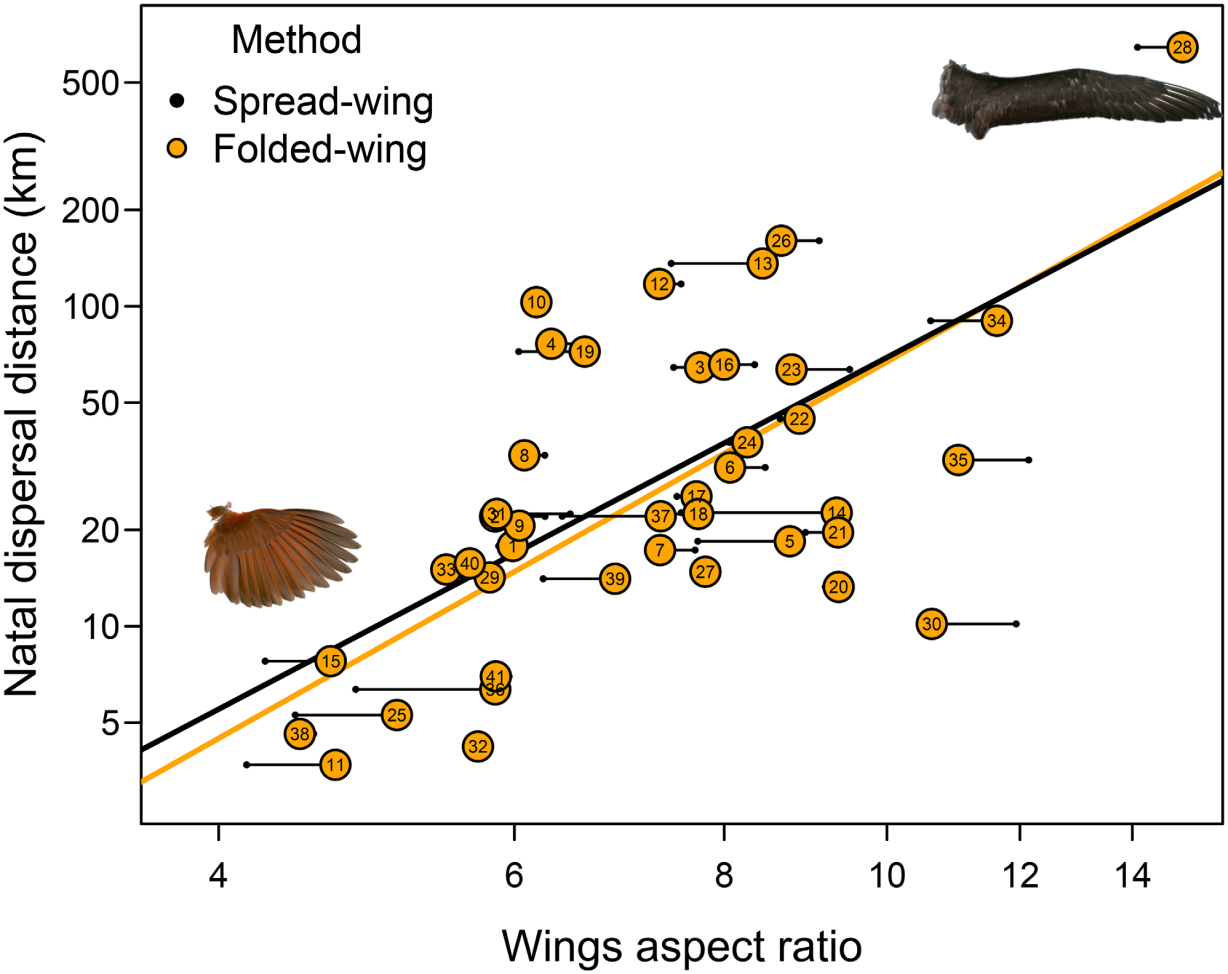
The relationship between the wing aspect ratio and natal dispersal distances among 41 species of North American birds. Aspect ratios were calculated using estimates of wing areas from the traditional spread‐wing method (small black dots) and from the new folded‐wing method (Ellipse model, orange dots). The black segments join the alternative estimates of the same species. Lines represent the corresponding phylogenetic regression lines. The numbers correspond to species as follows: (1) *Accipiter cooperii*, (2) *Accipiter gentilis*, (3) *Anas platyrhynchos*, (4) *Aquila chrysaetos*, (5) *Archilochus colubris*, (6) *Branta bernicla*, (7) *Branta canadensis*, (8) *Buteo jamaicensis*, (9) *Buteo lineatus*, (10) *Buteo regalis*, (11) *Cardinalis cardinalis*, (12) *Ardea alba*, (13) *Charadrius melodus*, (14) *Cygnus buccinator*, (15) *Dumetella carolinensis*, (16) *Falco peregrinus*, (17) *Falco sparverius*, (18) *Grus canadensis*, (19) *Haliaeetus leucocephalus*, (20) *Larus argentatus*, (21) *Larus californicus*, (22) *Larus delawarensis*, (23) *Larus marinus*, (24) *Pandion haliaetus*, (25) *Passerina cyanea*, (26) *Pelecanus erythrorhynchos*, (27) *Nannopterum auritum*, (28) *Phoebastria nigripes*, (29) *Dryobates borealis*, (30) *Rynchops niger*, (31) *Sialia currucoides*, (32) *Sialia mexicana*, (33) *Sialia sialis*, (34) *Sterna caspia*, (35) *Sterna hirundo*, (36) *Strix varia*, (37) *Tachycineta bicolor*, (38) *Thryothorus ludovicianus*, (39) *Tyto alba*, (40) *Zenaida asiatica*, (41) *Zenaida macroura*.

Wing aspect ratios calculated using the Ellipse‐Triangle model fitted data on natal dispersal distances of North American birds as well as wing aspect ratios calculated using the traditional spread‐wing method (Figure 5, Table 3). Differences in model fit were minimal.

**TABLE 3:** Phylogenetic regression coefficients and fit statistics for the relationship between the aspect ratio and natal dispersal distances among 41 North American bird species using the traditional spread‐wing method and the new folded‐wing method (Ellipse model) for estimating the total wing area.

Method	Intercept	Coefficient^a^	df	Log(Lik)^b^	AICc^c^	*t*	*p*	*r* ^2^
Spread‐wing Method	−2.12	2.76	2	−50.1	104.2	4.72	>.001	.36
Folded‐wing Method	−2.59	2.94	2	−50.2	104.4	4.61	>.001	.35

^a^ Aspect ratios and natal dispersal distances were log‐transformed.

^b^ Log(Lik) is the logarithm of the maximized likelihood.

^c^ AICc is the sample size corrected version of the Akaike information criterion.

## 4 DISCUSSION

In this study, we described and validated a new folded‐wing method for estimating the total wing area of birds. We found that the folded‐wing method produced total wing area estimates that were very similar to those produced by the traditional spread‐wing method but was more precise (showed a lower coefficient of variation). The increased precision of the new folded‐wing method is likely due to the fewer steps in which error can be introduced. In the traditional spread‐wing method, errors can be introduced during four procedural steps: (1) wing posturing and specimen preparation (e.g., under or over‐spreading the wing), (2) photographing (e.g., optical and perspective distortions), (3) digital processing (e.g., inaccuracies in setting the scale or image binarization), and (4) measuring distances. By contrast, the new method only requires measuring three distances: wingspan, wing length, and wing width. The wingspan is known to show relatively low repeatability (Winker, 1998), but wing length and wing width are highly repeatable even when measured by different researchers (Tobias et al., 2022).

Note that although wing areas estimated by the traditional spread‐wing method are a useful reference, they cannot be considered the “ground truth.” Because the avian wing is an articulated limb that can adopt different shapes and surface areas depending on the degree of flexion, there is no such thing as “the true wing area” that can be measured directly and objectively. Even minimizing all human‐related and tool‐related errors, the area estimated by the traditional method will depend on the posture of the wing. In this sense, the traditional method of estimating the surface area on a spread‐wing specimen is also a model, a model that assumes that the posture of the specimen measured is correct and the most relevant for aerodynamic purposes. That assumption does not hold for thousands of spread‐wing specimens that are either underextended or overextended, resulting in underestimation and overestimation of the relevant surface area.

The new method also fared well when used to predict empirical data on bird movement. The aspect ratio calculated using wing areas estimated with the folded wing method predicted natal dispersal distances of North American birds as well as the aspect ratio calculated using wing areas estimated with the traditional method. This demonstrates the effectiveness of the new method in studies of bird movement and dispersal.

**FIGURE 6:**
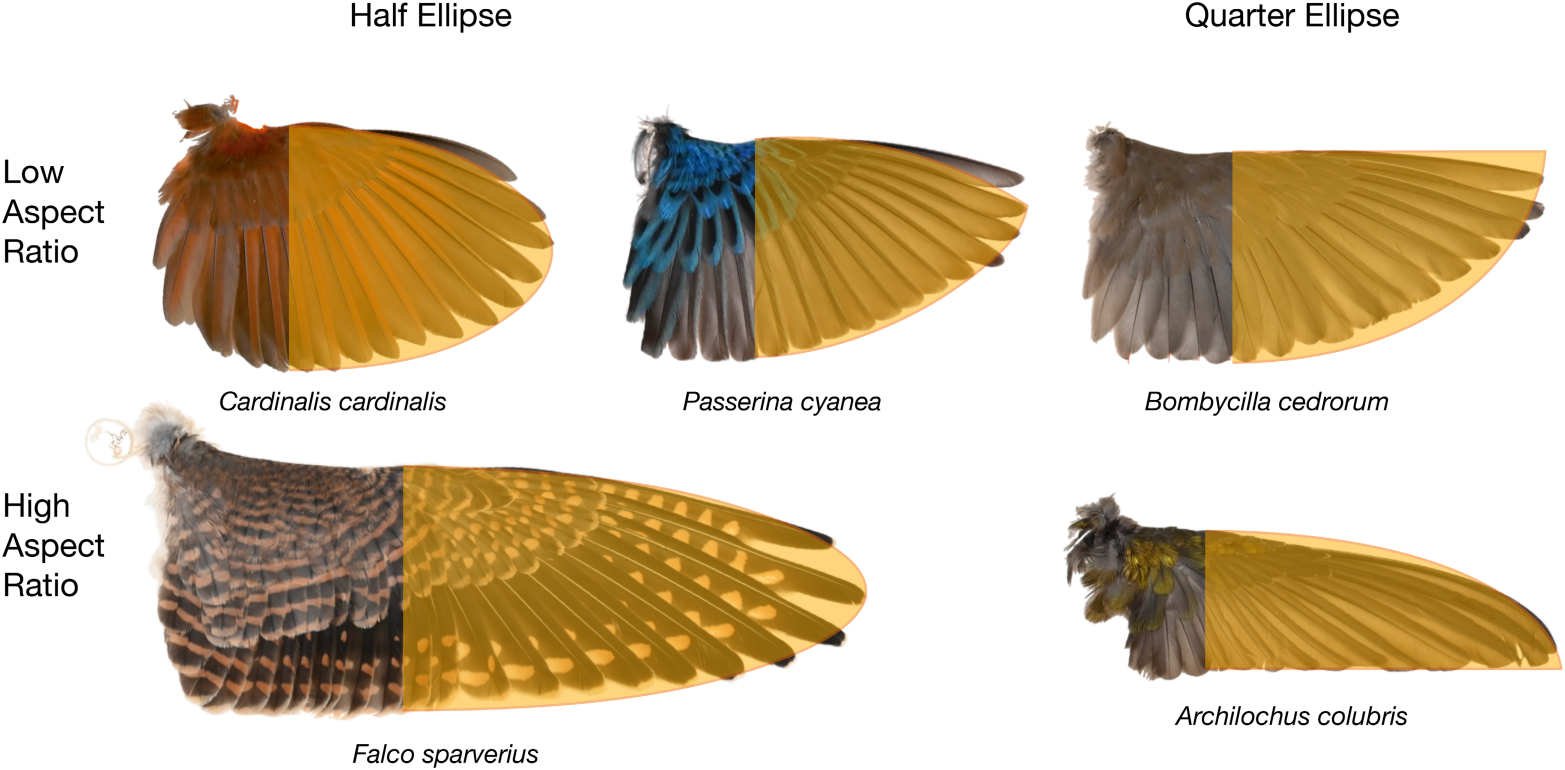
Spread‐wing specimens illustrating the fit of various forms of the Ellipse model including half ellipses (left), quarter ellipses (right), and a hypothetical intermediate shape that shares the same area formula (center). (*Cardinalis cardinalis* ROM513781, *Passerina cyanea* ROM513785, *Bombycilla cedrorum* ROM513799, *Falco sparverius* ROM513813, and *Archilochus colubris* ROM513825).

Among the geometric models evaluated for the folded‐wing method, assuming that the shape of the hand‐wing was a half or a quarter of an Ellipse resulted in more accurate and precise estimates as compared to assuming a triangular shape. Because assuming half or a quarter of an ellipse resulted in the same formula for the total wing area, this model has the potential to fit a wide variety of wing shapes. Birds with rounded wings such as jays, wrens and cardinals may be better represented by the half‐ellipse model, whereas species in which the most distal primaries are the longest such as waxwings, swallows, and gulls may be better represented by a quarter of an ellipse (Figure 5). In theory, there is an infinite number of shapes that lie somewhere in between the half‐ellipse and the quarter‐ellipse that would share the same area formula (Equation 3). Therefore, the Ellipse model has the potential to fit well a variety of wing shapes that are intermediate between a quarter of an ellipse and half of an ellipse (Figure 6).

Assuming that estimates from the traditional spread‐wing method are not biased, the simplest Ellipse model evaluated here showed a tendency to overestimate wing areas. This may be due to multiple factors and may depend on the shape of the wing. None of these simple geometrical models take into account the slots or gaps between feather tips, and the wings of many species may be less convex than what the elliptical curvature implies. We experimented with assuming that one of the sides of the wings was straight instead of convex, leading to the Ellipse‐Triangle model (Figure 2), and this modification reduced the bias to minimal levels. Therefore, while the basic Ellipse model provides a general estimator with minimal assumptions, we recommend the use of the Ellipse‐Triangle model for studies in which estimates from both spread wings and folded wings need to be combined.

It is possible that some shapes fit the wings of particular species better. For example, the Triangle model produces estimates closer to the spread‐wing method for *Stercorarius* (Figure 3b). However, there is not a clear trend for areas of pointy wings to be better estimated using the triangle model, and the same is true for other wing shapes and models (Figure 3b). However, interspecific model residuals have a strong phylogenetic structure (lambda = 1), suggesting that they do not represent just random error but phylogenetically structured error. Analyses of an expanded dataset will help determine if adding information on taxonomic identity or some aspect of wing morphology could potentially improve model fit.

Perhaps, the most critical advantage of the folded‐wing method, in addition to increasing precision, is the possibility of measuring wing areas without the need for an image of the extended wing. This opens the possibility for estimating the wing area of thousands of specimens in museum collections that have a measure of the wingspan recorded. For example, the Royal Ontario Museum has 19,949 study skin specimens with associated wingspan measurements (VertNet, http://vertnet.org). The use of old museum specimens will be particularly beneficial for obtaining estimates of wing area and aspect ratio for rare and poorly known species. In addition, the wing length and secondary length measurements for most bird species are already available in the AVONET database (Tobias et al., 2022). These measurements were originally collected for the purpose of calculating the hand‐wing index, which is a proxy for the hand‐wing aspect ratio. Given the historical limitation of obtaining total wing area estimates, the hand‐wing index has been extensively used as a proxy for aspect ratio and flight efficiency in studies of macroecology and evolution (Sheard et al., 2020; Tobias et al., 2020; Weeks et al., 2022). However, the actual aspect ratio and the lift‐to‐drag ratio are better proxies of long‐distance flight efficiency (Chu & Claramunt, 2023; Claramunt, 2021). The new folded‐wing method will make possible the use of these better proxies of flight efficiency in large‐scale comparative analyses of bird flight and dispersal in birds.

Facilitating the estimation of flight performance and dispersal abilities can have important implications for bird conservation biology (Claramunt et al., 2022; Travis et al., 2013; Van Houtan et al., 2010). One key application of the folded‐wing method is contributing to assessing species' vulnerability to anthropogenic impacts. The current rapid decline of avian populations places urgency on mitigating an even steeper decline in the near future. Two major threats to birds, habitat fragmentation and climate change, are expected to affect species with limited ability to move more severely (Lens et al., 2002; Şekercioḡlu et al., 2002; Sodhi et al., 2004). The new folded‐wing method can help identify species with low dispersal capabilities that would be more vulnerable to habitat fragmentation and climate change. We cannot bring species back from extinction, but quantifying the dispersal limitation of bird species may be crucial in assessing their extinction risk and defining targets for conservation efforts.

## AUTHOR CONTRIBUTIONS

**Hellen (Yi) Fu:** Conceptualization (equal); data curation (lead); formal analysis (lead); writing – original draft (lead). **Michelle Su:** Data curation (supporting); writing – review and editing (supporting). **Jonathan J. Chu:** Data curation (supporting); resources (supporting); writing – review and editing (supporting). **Alexandra Margaritescu:** Data curation (supporting); writing – review and editing (supporting). **Santiago Claramunt:** Conceptualization (equal); formal analysis (supporting); funding acquisition (lead); supervision (lead); writing – original draft (supporting).

## FUNDING INFORMATION

This study received support from the Natural Sciences and Engineering Research Council of Canada (NSERC) Discovery Grant RGPIN‐2018‐06747 to S.C.

## Supporting information


Appendix 1.

Appendix 2.

## Data Availability

Morphometric data collected during this study are available in Appendices 1 and 2. Images of spread‐wing specimens used in the analyses are available in the Dryad data repository (https://doi.org/10.5061/dryad.cc2fqz6c9).
